# Antibody and B cell responses to *Plasmodium* sporozoites

**DOI:** 10.3389/fmicb.2014.00625

**Published:** 2014-11-18

**Authors:** Johanna N. Dups, Marion Pepper, Ian A. Cockburn

**Affiliations:** ^1^Department of Pathogens and Immunity, John Curtin School of Medical Research, Australian National UniversityCanberra, ACT, Australia; ^2^Department of Immunology, School of Medicine, University of WashingtonSeattle, WA, USA

**Keywords:** malaria, *Plasmodium*, B cells, antibodies, sporozoites, pre-erythrocytic stages

## Abstract

Antibodies are capable of blocking infection of the liver by *Plasmodium* sporozoites. Accordingly the induction of anti-sporozoite antibodies is a major aim of various vaccine approaches to malaria. In recent years our knowledge of the specificity and quantities of antibodies required for protection has been greatly expanded by clinical trials of various whole sporozoite and subunit vaccines. Moreover, the development of humanized mouse models and transgenic parasites have also aided our ability to assess the specificity of antibodies and their ability to block infection. Nonetheless, considerable gaps remain in our knowledge – in particular in understanding what antigens are recognized by infection blocking antibodies and in knowing how we can induce robust, long-lived antibody responses. Maintaining high levels of circulating antibodies is likely to be of primary importance, as antibodies must block infection in the short time it takes for sporozoites to reach the liver from the skin. It is clear that a better understanding of the development of protective B cell-mediated immunity will aid the development and refinement of malaria vaccines.

## INTRODUCTION

The generation of protective antibodies underpins the success of almost all of our current vaccines (Plotkin, 2008, 2010). While there is no licensed vaccine available for malaria, one indication that a vaccine may be achievable comes from the seminal finding that immunization with radiation attenuated sporozoites (RAS) results in protection against live parasite challenge in both mice and humans (Nussenzweig et al., 1967; Clyde et al., 1973; Hoffman et al., 2002; Seder et al., 2013). Moreover, complete protection against malaria has also been demonstrated in volunteers immunized with low numbers of infectious bites under chloroquine prophylaxis (Roestenberg et al., 2009), also known as DAP immunization. Protective responses induced by RAS appear to be principally based on CD8^+^ T cells and antibodies (Schofield et al., 1987; Weiss et al., 1988), with contributions from CD4^+^ T cells, gamma-delta T cells and natural killer (NK) cells also proposed (Tsuji et al., 1994; Doolan and Hoffman, 1999; Oliveira et al., 2008). Antibodies could block infection at the pre-erythrocytic stages in several ways, either by neutralizing sporozoites directly, opsonizing sporozoites for phagocytosis or blocking invasion of parasites into hepatocytes. There is also some evidence that antibodies can block liver stage development, though the mechanism for this is unclear (Chatterjee et al., 1996). In this minireview we will examine the evidence that antibodies can play important roles in protection, evaluate the targets of those antibodies and determine what needs to be known to advance our knowledge of antibody and B cell immunity to sporozoites, and potentially contribute to vaccine development.

## ANTIBODY MEDIATED PROTECTION AGAINST SPOROZOITE CHALLENGE

### EARLY STUDIES ON HUMORAL IMMUNITY: THE IDENTIFICATION OF THE CIRCUMSPOROZOITE PROTEIN AS A TARGET OF PROTECTIVE IMMUNITY

Shortly after the discovery of protective immunization with RAS, it was found that RAS immunized mice rapidly cleared the sporozoite inoculum from circulation, suggesting the presence of neutralizing antibodies (Nussenzweig et al., 1972). Later transfer experiments showed that immunoglobulin G (IgG) and T cells acted synergistically to confer sterile immunity to sporozoites, (Schofield et al., 1987). More recently, sera from individuals immunized with DAPs was found to reduce infection of humanized mice infected with *Plasmodium falciparum* (Behet et al., 2014). Other early evidence for a role of antibodies in protection came from the identification of mAbs capable of inducing the precipitation of material from the surface of human and rodent malaria sporozoites – a phenomenon known as the circumsporozoite reaction (Yoshida et al., 1980). These mAbs were shown to be capable of blocking infection *in vitro* (Nardin et al., 1982), and *in vivo* (Potocnjak et al., 1980). Subsequently the target of these antibodies was cloned and identified as the CSP (Ellis et al., 1983; Dame et al., 1984; Enea et al., 1984)

CSP is a GPI-anchored protein consisting of a conserved domain structure with N- and C-terminal domains separated by an asparagine-rich repeat region. The C-terminal domain contains a conserved TSR, which is important for the recognition and binding of hepatocytes (Cerami et al., 1992; Frevert et al., 1993). The N-terminal domain acts by masking the TSR of the C-terminal domain, and has to be cleaved to allow the parasite to invade hepatocytes (Coppi et al., 2011). In contrast, the role of the repeat region, which in *P. falciparum* consists of (NANP)_n_ repeats with a few NVDP repeats interspersed at the beginning, is unknown. Nonetheless, this region was identified early on as an important target of protective immunity, and contains the epitopes recognized by all the original anti-CSP mAbs reported (Zavala et al., 1983, 1985). In terms of protective immunity, much less work has been done to investigate antibody responses to the N- and C-terminal domains despite their functional importance. Several studies have shown that immunization with N-terminal peptides can induce invasion-blocking antibodies (Rathore et al., 2005; Bongfen et al., 2009). Interestingly, a correlation between the presence of antibodies to this region with a reduction in malaria morbidity has also been observed (Bongfen et al., 2009).

### PROTECTION MEDIATED BY ANTI-CSP ANTIBODIES IN HUMANS: LESSONS FROM VACCINE TRIALS

Perhaps, the strongest evidence that anti-CSP antibodies can be protective comes from trials of the CSP-based RTS,S vaccine (Stoute et al., 1997). RTS,S is a virus-like particle consisting of 19 NANP repeats and C terminal domain of the CSP fused to the Hepatitis B Surface antigen. RTS,S is currently in Phase III clinical trials in a formulation with AS01, a proprietary adjuvant consisting of a mixture of liposomes, monophosphoril lipid A and saponin (Casares et al., 2010). In experimental challenges of malaria-naïve adults, RTS,S gives short-lived sterile protection in around 50% of volunteers (Kester et al., 2001, 2009). In phase III clinical trials in endemic areas, RTS,S gave 56% protection against clinical malaria among 5–17 month old children (Agnandji et al., 2011b), and 31% efficacy among younger infants (Rts et al., 2012). While there is some evidence of reduced numbers of infections in the field (Guinovart et al., 2009), the main effect of the vaccine appears to be on disease severity, which is surprising as CSP is not expressed in the pathogenic blood stages. The data are however similar to the findings of Bongfen et al. (2009; described above) showing protection against disease correlating with high titres of N-terminus specific antibodies. One explanation for these results is that the vaccine might lower the initial inoculum of parasites and thus the number of blood stages emerging from the liver, buying time for the immune system to control infection. It may also be that while the vaccine does not block all infectious bites, the breakthrough infections are less likely to be genetically complex or highly virulent (Moorthy and Ballou, 2009). Importantly, these data rebut one of the traditional objections to pre-erythrocytic stage vaccines, namely that they would be ineffective if parasites do breakthrough and establish blood stage infection. Nonetheless, this does not negate the importance of developing vaccines targeting other life cycle stages in tandem with pre-erythrocytic approaches.

The mechanisms of protection by RTS,S are poorly understood, with different trials measuring different immunological parameters. Most studies report ELISA titres of total IgG responses against the (NANP)_n_ repeat, but some use μg/ml while other report titres as ELISA Units (EU). Only in early studies were antibodies segregated by subclass, with no association reported between subclasses and protection (Stoute et al., 1997; Kester et al., 2001). Antibodies to the C-terminal domain have been little studied and were not found to be associated with protection (Kester et al., 2001). Finally only one study reports the number of CSP-specific B cells in vaccinated individuals (Agnandji et al., 2011a). Nonetheless direct evidence that RTS,S induced antibodies can protect comes from a recent study in which human mAbs targeting the CSP repeat were shown to block *P. falciparum* infection of humanized mice (Foquet et al., 2014).

Clinical trials of the vaccine both in naïve individuals and in the field also provide strong evidence of a role for anti-CSP antibodies. Mathematical modeling of a Phase IIb trial in which malaria naïve volunteers were given the RTS,S vaccine formulated in either AS01B or AS02A (Kester et al., 2009), suggested that the bulk of protection comes from high levels (100–200 μg/ml) of anti-repeat antibodies, aided by robust CD4^+^ T cell responses (White et al., 2013). Importantly, in this study protection did not correlate with titres of Hepatitis B antibodies – which are also induced by the vaccine – suggesting that the CSP antibodies were mediating protection and were not merely a correlate of vaccine “take” (Kester et al., 2009). In endemic areas, mathematical modeling based on a meta-analysis of all Phase II trials has suggested a threshold for infection blocking immunity around 51 EU/ml among children and infants, which is probably similar to the levels required for protection in naïve volunteers (White et al., 2014).

### ANTI-CSP ANTIBODIES: OUTSTANDING QUESTIONS

Regardless of the exact measurement used, it is clear that very high titres of antibody are required for protection. While it is difficult to compare directly with other vaccine regimens, the protective cutoff for vaccines to *Haemophilus influenzae* and *Pneumococcus* are <1 μg/ml (Plotkin, 2008), which leads us to ask, why so much antibody is required for protection against sporozoites? One possible answer is that a high amount of antibody is required to eliminate every sporozoite in the short time it takes for the parasites to exit the skin and migrate to the liver. Nonetheless it is not clear how many of the antibodies measured by ELISA are functional: it may be that only the highest affinity antibodies are capable of sporozoite neutralization. Interestingly, in trials of RAS or DAP, *in vivo* protection and *in vitro* infection blocking is obtained at quite low anti-CSP titres (Seder et al., 2013; Behet et al., 2014; Finney et al., 2014). This may be because these whole parasite vaccines stimulate a broad range of protective immune responses, but it is also possible that they induce antibodies that are better able to recognize the native conformation of CSP. Finally there has been no biophysical or structural characterization of the binding of anti-CSP antibodies. These data would give an idea of the necessary affinity required for sporozoite neutralization and show how antibodies bind to the repeat region, which is likely to be somewhat disorganized (Plassmeyer et al., 2009) – it seems likely that these antibodies may have to stabilize the CSP structure and thus pay a high “entropic cost” in binding.

### THE ROLE OF OTHER ANTIGENS IN PROTECTION

The limited success of CSP-based recombinant vaccines relative to whole parasite approaches has led to a search for other targets of anti-sporozoite antibodies. Immunity to CSP is probably not absolutely required for protection in rodent models as shown by experiments in which mice immunized with wild type *P. berghei* RAS were fully protected against challenge with *P. berghei* sporozoites expressing CSP from either *P. yoelii* or *P. falciparum* (Gruner et al., 2007; Mauduit et al., 2009). Nonetheless, it is unclear how much of this CSP-independent protection is mediated by antibodies rather than T cells.

A variety of potential protective antigens have been identified in the sporozoite and liver stages by classical approaches (Duffy et al., 2012). However, while T cell responses to many of these antigens such as LSA-1 and TRAP are relatively well studied (Duffy et al., 2012; Offeddu et al., 2012), the evidence that antibodies to these proteins may be protective is limited (**Table 1**). Combined antibody titres to CSP, LSA-1 and TRAP have been correlated with reduced incidence of clinical malaria among Kenyan children (John et al., 2005), while antibodies to LSA-1 alone have been associated with protection from reinfection following treatment (Domarle et al., 1999). Early reports suggested that antibodies targeting STARP could have even stronger invasion blocking activity than anti-CSP antibodies, there has been little further examination of this molecule (Fidock et al., 1997). Nonetheless when TRAP and LSA-1 were formulated as vaccines in combination with the AS01 or AS02 adjuvant, they were unable to elicit protection (Cummings et al., 2010; Kester et al., 2014).

**Table 1:** Potential non-CSP targets of anti-sporozoite antibodies.

Antigen	Evidence	Reference
TRAP/SSP2	Antibodies associated with protection from infection in endemic area Mouse monoclonal antibodies display modest infection blocking activity *in vitro*	John et al. (2005) Charoenvit et al. (1997)
LSA-1	Antibodies associated with protection from infection in endemic area LSA-1 repeat antibodies correlate with protection from reinfection in a drug treated cohort	John et al. (2005) Domarle et al. (1999)
STARP	Affinity purified antibodies from exposed individuals block sporozoite invasion *in vitro*	Fidock et al. (1997)
MB2	Rabbit polyclonal sera inhibit sporozoite invasion *in vitro* Antibody levels correlate with protection in RAS immunized volunteers	Nguyen et al. (2009) Nguyen et al. (2009)

More recent studies have used protein microarrays to examine the diversity of antibody responses induced after RAS or DAP immunization of humans (Trieu et al., 2011; Felgner et al., 2013). Antibody profiling of RAS vaccines by protein microarray revealed strong antibody responses to two other established vaccine candidates (AMA1 and TRAP) in addition to CSP and a large number of proteins that had not previously been associated with protection. Many of the remaining proteins were hypothetical or involved in cell cycle functions. Protein microarray analysis of DAP immunized volunteers revealed a different antibody profile with CSP and LSA-1 being the only antigens recognized by all protected individuals. Overall, these studies suggest that no single molecule is the key to protection, rather protective antibody responses consist of broad responses to numerous antigens.

## B CELL RESPONSES TO *Plasmodium* SPOROZOITES

**FIGURE 1:**
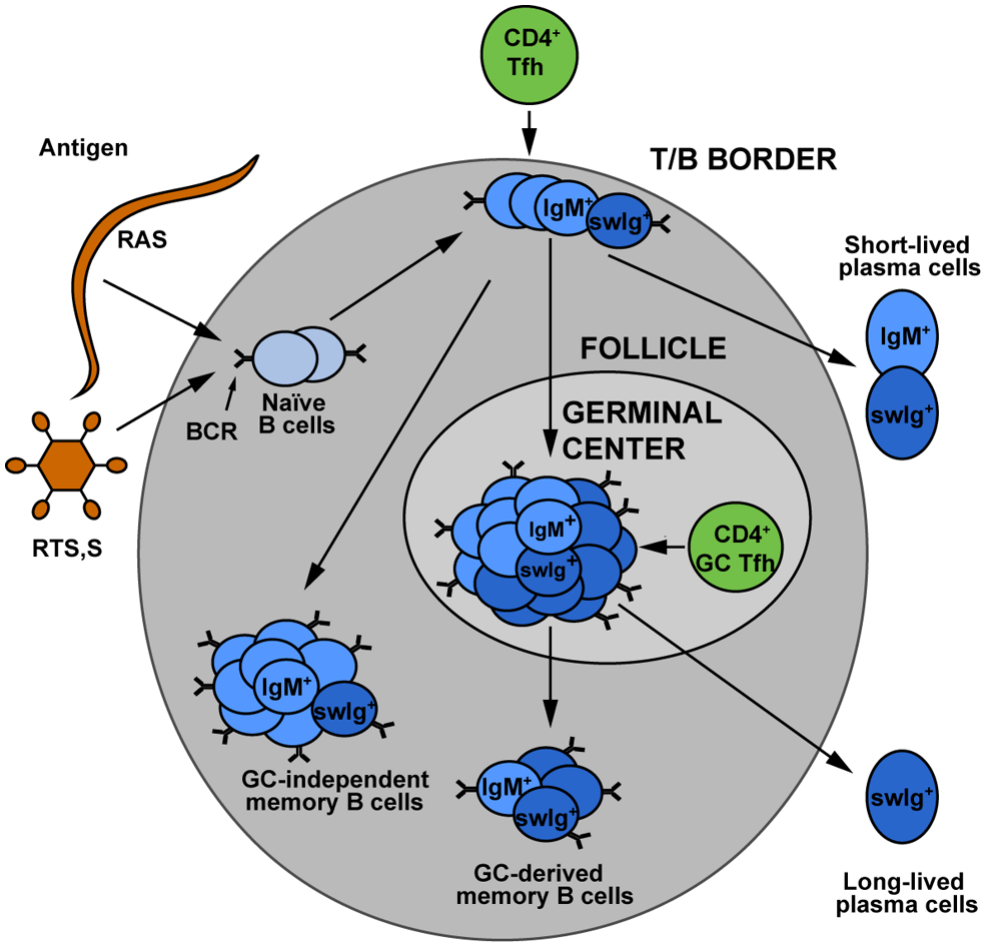
**The development of memory B cell subsets.** Upon encounter with either sporozoite antigen or vaccines, naïve B cells can undergo a variety of different fates. Some develop into short-lived plasmablasts, which give an immediate antibody response to infection. Others may become “early memory” which is germinal center independent, or may enter germinal centers where theirB cell receptors undergo somatic hypermutation and affinity maturation. The germinal center B cells are believed to be the major precursors for long-lived plasma cells, which maintain the circulating antibody pool. Memory cells may be class switched or they may retain the IgM^+^ B cell receptor. The relative contributions of these different memory populations to long term protection against malaria remains an area for further investigation. Figure is based on Taylor et al. (2012). Used with permission from Elsevier.

While vaccination studies in particular have provided insight into protective antibody responses to sporozoites, sporozoite-specific B cell memory and plasma cell formation is poorly understood. Recognition of a foreign antigen by a B cell receptor leads to the proliferation and differentiation of the activated B cell resulting in the formation of short-lived plasma blasts, “early memory” B cells (that do not enter germinal centers) and germinal center B cells (Zotos and Tarlinton, 2012). Germinal center B cells are generally considered the precursors of LLPCs that can maintain antibody titres (**Figure 1**). Knowledge of the development and maintenance of LLPCs would be of particular interest as the rapid transit of sporozoites from skin to liver offers little or no opportunity for anamnestic responses (memory B cells) to contribute to protection. To the best of our knowledge, no work has been performed in animal models to examine the development and longevity of sporozoite specific B cells and plasma cells. There are however a number of observations of sporozoite specific B cells in naturally exposed and DAP or RTS,S vaccinated individuals. A study in Thailand reported very low levels of B cell responses following natural exposure with only 1/33 adults having CSP-specific B cells (Wipasa et al., 2010). More robust responses are seen following immunization: CSP specific cells accounted for 1% of circulating IgG secreting B cells following RTS,S vaccination and 0.25% of circulating IgG secreting B cells after DAP treatment (Agnandji et al., 2011a; Nahrendorf et al., 2014). These studies are limited however, in that they rely on restimulation enzyme-linked immunospot (ELISPOTs) and therefore cannot provide information on cell phenotype, they report over relatively short timescales and they investigate only IgG antibody secreting cells. Many of the shortcomings of the ELISPOT approach could be overcome by the use of fluorescently labeled antigens to detect rare B cell populations by flow cytometry, however while this has been performed previously for blood stage antigens (Muellenbeck et al., 2013), no such studies have yet been performed with sporozoite antigens.

### THE ACTIVATION OF SPOROZOITE SPECIFIC MEMORY B CELLS

A key outstanding question is how are B cells primed by sporozoites? This is particularly important given that immunization with RAS and DAP represent our most successful immunization approaches. It has been shown that after mosquito biting, a large proportion of parasites remains in the skin and a subset of these migrate to the draining lymph node (Amino et al., 2006). The skin draining lymph node appears to be an important location for the induction of protective immunity to sporozoites: not only is it the first location where sporozoite specific CD8^+^ T cells are detected, it has also been shown that the removal of this lymph node along with the spleen completely abrogates RAS-mediated protection (Chakravarty et al., 2007). By extension it seems likely that the first interactions of B cells with sporozoite antigens occur at this site.

The role of CD4^+^ T cells, and in particular T follicular helper cells in providing help for antibody responses is also a neglected area. Sporozoites can induce CD4^+^ T cells and numerous CD4^+^ epitopes have been identified in the CSP proteins of both mice and human malaria strains (Nardin and Nussenzweig, 1993). Moreover, immunization studies with multiple antigen peptides have shown that B cell responses to the NANP repeat are enhanced by the inclusion of T cell epitopes (Tam et al., 1990). CSP-specific CD4^+^ T cells expressing various effector functions area are also associated with protection by RTS,S (White et al., 2013). Nonetheless the extent to which these cells are acting as direct effectors or through help to antibody responses is unclear.

### THE INFLUENCE OF BLOOD STAGE MALARIA ON B CELL RESPONSES TO SPOROZOITES

One critical factor that may affect the maintenance of sporozoite specific immunity, and immunity to vaccines in general, is malaria infection itself (Urban et al., 1999; Ocana-Morgner et al., 2003). In mouse models, the impact of blood stages on both bystander and malaria-specific immune responses has been examined. *P. yoelii* infection induces apoptosis of memory B cells and plasma cells specific for the blood stage antigen MSP-1 (Wykes et al., 2005); interestingly however, *P. yoelii* infection also induced apoptosis of bystander plasma cells. A similar effect has been reported for Influenza A-specific plasma cells following infection with *P. chabaudi* (Ng et al., 2014). This suggests that blood stage infection may cause a generalized loss of plasma cells and memory B cells irrespective of their specificity (Wykes et al., 2005). It is reported that this apoptotic effect is the result of decreased levels of B cell survival factor (BAFF) expression by conventional dendritic cells in infected mice (Liu et al., 2012). In humans, BAFF expression was found to increase during acute malarial infection and is associated with more severe disease rather than less (Nduati et al., 2011). The rise in soluble BAFF is also correlated with a general proliferation of B cells in volunteers given a controlled malaria challenge (Scholzen et al., 2014).

Blood stage malaria infections in humans have also been associated with high levels of so-called atypical memory B cells (Weiss et al., 2009, 2011; Portugal et al., 2012; Illingworth et al., 2013). Atypical memory B cells, characterized by low expression of CD21 and CD27, have also been described in HIV-infected viremic patients and display exhausted/anergic behavior (Moir et al., 2008). Although they exhibit an ‘exhausted’ phenotype in malaria infection by displaying decreased *in vitro* ability to differentiate upon stimulation into plasma cells (Weiss et al., 2009), atypical memory B cells isolated from asymptomatic semi-immune donors appear to be functional and may secrete anti-*P. falciparum* IgG (Muellenbeck et al., 2013). It would be desirable to know if sporozoite-specific B cells are driven to form atypical memory, either as bystanders to blood stage infection or due to continued antigen exposure – e.g., from frequent biting, or cross reactivity with blood stage antigens.

## CONCLUDING REMARKS

Together the available data tell us that anti-sporozoite antibodies can protect and should be a major component of a pre-erythrocytic vaccine. Beyond CSP, however the targets of protective immunity are unknown. Further vaccine development is also hampered by a lack of basic knowledge on such issues as the optimal fine specificity, affinity and subclass required for protection by anti-sporozoite antibodies. Finally a successful vaccine will have to induce longer-lived plasma cell and memory responses than existing candidates. To understand these issues a better understanding of the immunology of anti-sporozoite B cell responses seems essential; such knowledge may enable the development of new subunit approaches, or enable us to optimize whole parasite vaccines.

## AUTHOR CONTRIBUTIONS

Johanna N. Dups and Ian A. Cockburn jointly wrote the first draft of this manuscript. Marion Pepper reviewed the manuscript and contributed significantly to the final draft. All authors read and approved the final manuscript.

## Conflict of Interest Statement

The authors declare that the research was conducted in the absence of any commercial or financial relationships that could be construed as a potential conflict of interest.

## Abbreviations

AMA-1: apical membrane antigen 1
CSP: circumsporozoite protein
DAP: drug arrested parasites
LLPC: long-lived plasma cell
LSA-1: liver stage antigen 1
mAb: monoclonal antibody
STARP: serine threonine and asparagine rich protein
TRAP: thrombospondin repeat anonymous protein
TSR: thrombospondin repeat
